# Takotsubo Syndrome After Pacemaker Implantation: A Case Report and Literature Review

**DOI:** 10.19102/icrm.2024.15051

**Published:** 2024-05-15

**Authors:** Elia De Maria, Ambra Borghi, Michele Mario Cinelli, Vittorio Topazio, Stefano Cappelli, Jonathan Galloni, Giuseppe Boriani

**Affiliations:** 1 Cardiology Unit, Ramazzini Hospital, Carpi, Modena, Italy; 2 Cardiology Division, Department of Biomedical, Metabolic and Neural Sciences, University of Modena and Reggio Emilia, Policlinico di Modena, Modena, Italy

**Keywords:** CIED complications, pacemaker implantation, takotsubo syndrome

## Abstract

A 78-year-old male patient with complete atrioventricular block underwent an uncomplicated pacemaker implantation. After 24 h, he presented acute chest pain, dyspnea, ST-segment–elevation in the anterior leads, left ventricular apical ballooning, and an ejection fraction of 35%. His coronary angiogram was normal. Within 2 days, his symptoms and electrocardiogram (ECG) abnormalities disappeared, while wall motion abnormalities recovered after 6 weeks. A diagnosis of takotsubo syndrome (TTS) was made. Pacemaker implantation has been described as a potential trigger for TTS. The clinical picture exhibits some peculiarities, including a higher percentage of men and asymptomatic patients and challenging ST-segment interpretation of paced ECGs. It is unclear whether pathophysiologic mechanisms are different compared to other forms of TTS and whether the acute initiation of ventricular pacing plays a role.

## Introduction

Takotsubo syndrome (TTS) is an acute cardiac condition characterized by transient wall motion abnormalities, usually left ventricular (LV) apical ballooning, mimicking acute coronary syndrome (ACS), with a prevalence of 1%–3% among all patients presenting with ST-segment–elevation ACS.^1^ TTS has been typically reported after emotional or physical trigger events; however, several alternative mechanisms and different stimuli can elicit it. Pacemaker implantation has been described as a potential trigger for TTS, and its prevalence is not so rare and likely underestimated, also considering that about 20% of patients are mildly or entirely asymptomatic.^2^ The clinical picture in this setting shows some peculiarities, and it is still unclear whether pathophysiologic mechanisms are somehow different compared to other forms of TTS and whether the acute initiation of right ventricular (RV) pacing is a possible trigger. In this article, after providing a description of a case that occurred in our center, we review and discuss currently available knowledge about this diagnosis, which is worthy of consideration in clinical practice.

## Case presentation

**Figure 1:**
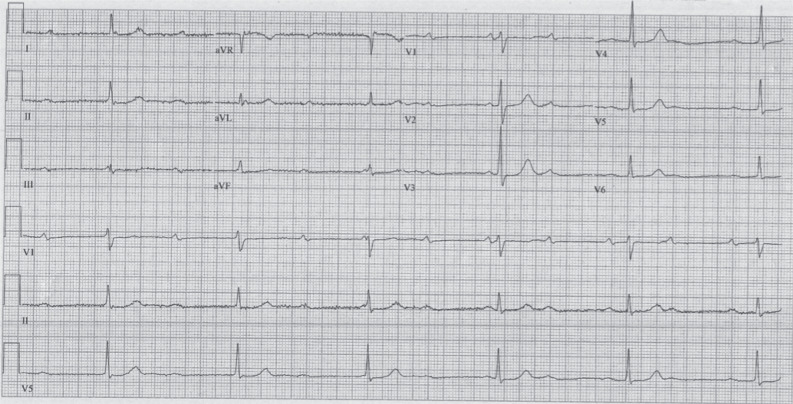
Electrocardiogram taken at admission showing third-degree atrioventricular block with narrow QRS junctional escape rhythm at 35 bpm (25 mm/s, 10 mm/mV, 40 Hz).

**Figure 2:**
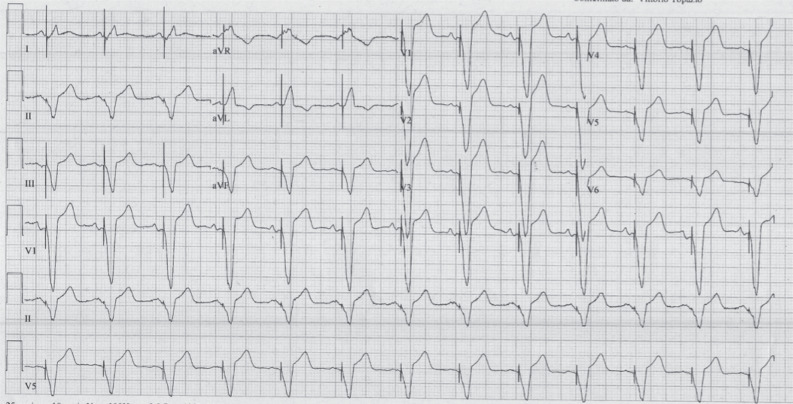
Postprocedural electrocardiogram showing atrial-tracked ventricular paced rhythm at 70 bpm with moderate ST-segment–elevation in precordial leads (maximum 3–4 mm) that is usually seen during pacing from the right ventricular apex (25 mm/s, 10 mm/mV, 100 Hz).

A 78-year-old man was admitted to our cardiology unit for severe bradycardia associated with fatigue and dizziness. His medical history was characterized by hypertension, hypercholesterolemia, impaired fasting glucose, and grade 3 obesity (height, 177 cm; weight, 140 kg; body mass index, 44.6 kg/m^2^). His medical treatment was 80 mg of telmisartan and 12.5 mg of hydrochlorothiazide daily. On admission, his vital signs were stable, his temperature was 36.5°C, his heart rate was 35 bpm, his respiratory rate was 18 breaths/min, and his blood pressure (BP) was 145/85 mmHg. Results of the physical examination were normal. Results of routine blood tests, including for liver, renal, and thyroid function; electrolytes; B-type natriuretic peptide; and troponin T levels were within the normal ranges. The electrocardiogram (ECG) on admission **(Figure 1)** showed third-degree atrioventricular (AV) block with narrow QRS junctional escape rhythm at 35 bpm. Echocardiography revealed mild LV concentric hypertrophy, an LV ejection fraction (LVEF) of 60%, mild mitral regurgitation, and a small posterior pericardial effusion. Following exclusion of reversible causes of AV block, the patient underwent implantation of a dual-chamber pacemaker, with atrial and ventricular leads inserted through the left cephalic vein. Isoproterenol administration was not administered prior to or during the implantation. During the procedure, the patient was greatly agitated and complained of pain at the site of the pocket, despite generous use of local anesthesia (lidocaine 2%) and intravenous midazolam. The duration of the procedure was longer than usual because of some difficulties experienced in crossing the tricuspid valve and in placing the RV lead. His-bundle and left bundle branch (LBB) pacing were attempted but not successful. In the end, the lead was placed in the RV apex with acceptable electrical parameters, and the procedure was concluded without complications. Postprocedural ECG **(Figure 2)** showed an atrial-tracked, ventricular-paced rhythm at 70 beats/min, with moderate ST-segment–elevation in precordial leads (3–4 mm) as usually seen during pacing from the RV apex.

**Figure 3:**
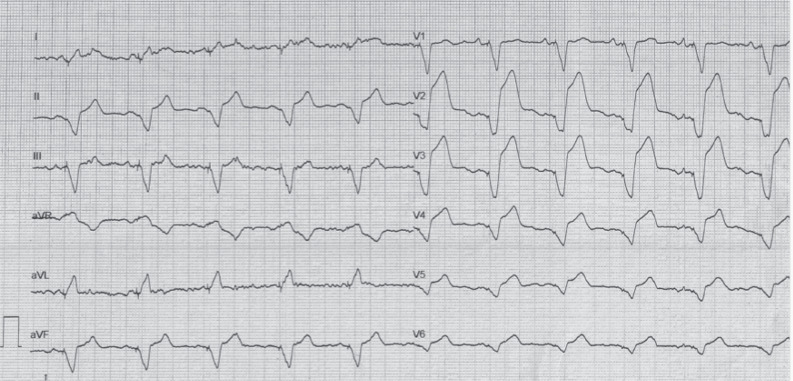
Electrocardiogram taken at the time of acute symptoms 24 h after implantation showing an atrial-tracked ventricular paced rhythm at 65 bpm with significant ST-segment elevation from V2–V5 leads (V2: 8 mm, V3: 6 mm) and diffuse attenuation of QRS amplitude, especially in lead V6 (25 mm/s, 10 mm/mV, 100 Hz).

However, 24 h after the procedure, the patient complained of acute chest pain and shortness of breath. Physical examination was notable for rales in the mid-basal lung fields, his BP was 160/95 mmHg, and his oxygen saturation was 92% on room air. An ECG taken during symptoms showed a ventricular paced rhythm with significant ST-segment–elevation from V2–V5 (8 mm in V2 and 6 mm in V3) as well as widespread attenuation of QRS amplitude more evident in lead V6 **(Figure 3)**. Echocardiography revealed new onset of apical akinesis and ballooning, an LVEF of 35%, septal dyskinesis, and no LV outflow tract obstruction; his pericardial effusion remained unchanged. Aspirin, metoprolol, furosemide, and nitrate were administered with benefit. The troponin level was significantly increased (peak value > 13,000 ng/mL, normal value < 20 ng/mL). The patient underwent coronary angiography, which revealed no significant coronary artery stenosis; ventriculography was not performed. Thereafter, a chest computed tomography scan was carried out, which excluded cardiac perforation. After 2 days, ECG abnormalities disappeared, and symptoms and signs of congestion resolved; LV apical hypokinesis persisted with the LVEF mildly improved to 40%–45%. Pacemaker electrical parameters were good, with a 100% paced rhythm. The patient was discharged 7 days after pacemaker implantation on 100 mg of aspirin, 80 mg of telmisartan, 100 mg of metoprolol, 25 mg of furosemide, and 50 mg of canrenone. At his 8-week follow-up visit, the patient was in good clinical condition, his ECG remained stable (nearly 100% ventricular paced rhythm), echocardiography showed an LVEF of 55%–60%, and contractility of the LV apex was normal, with only septal dyskinesis persisting.

Written informed consent was obtained from the patient for the publication of this case report, including accompanying images.

## Discussion

TTS is typically reported after emotional or physical trigger events and is usually characterized by transient LV wall motion abnormalities mimicking ACS, with a prevalence of 1%–3% among patients presenting with ST-segment elevation.^1^ The exact pathophysiologic mechanism remains unclear but likely involves an acute toxic effect of elevated blood levels of catecholamines and increased activity of sympathetic stimulation of the heart. Moreover, endothelial dysfunction, metabolic abnormalities, microvascular vasospasm, and a low level of circulating estrogen (in women) can play a role. The increasing complexity in the assessment of TTS has prompted the development of standardized diagnostic criteria, such as those proposed by an international consensus (InterTAK Score).^1^

Beyond classical triggers, pacemaker implantation can be responsible for TTS. Kurisu et al.^3^ were the first, in 2006, to describe pacing-induced TTS in two elderly women with AV block undergoing uncomplicated device implantation without previous isoproterenol infusion. These authors hypothesized a possible pathogenetic role of RV pacing combined with the stress of the procedure in an acute setting of severe bradyarrhythmia.

Since then, several other cases have been reported. Recently, Strangio et al.^2^ performed a systematic review of the literature in 2022. In their paper, they selected studies that fulfilled predefined inclusion criteria, including occurrence shortly after pacemaker implantation, the presence of apical ballooning, and no obstructive coronary artery disease, with other causes ruled out or considered unlikely. Studies were excluded if the diagnosis of TTS was not confirmed or not sufficiently discussed according to the InterTAK Score^1^ and if the relationship with pacemaker implantation was not clear. Selected articles included 17 case reports and 1 monocentric registry, with a total of 28 patients (21 women, 7 men) with a mean age of 74 years (range, 0–91 years). In 19 cases, the indication for pacemaker implantation was AV block or atrial fibrillation with slow ventricular response; sick sinus syndrome (SSS) was reported in the remaining 9 cases. Most of the devices (23/28) were dual-chamber pacemakers. It is interesting to note that male patients totaled 25% (n = 7) in the study by Strangio et al.^2^ compared to 9%–10% in the unselected TTS population.^1^ ECG changes were recorded in almost all cases, except for a few patients in whom ventricular pacing masked ST-segment abnormalities. Most patients were symptomatic, with symptoms appearing between 10 min and 3 days after the procedure; in 86% of cases, symptoms developed in the first 24 h. The majority of patients reported dyspnea, chest discomfort, orthopnea, nausea, and light-headedness. Some had hypotension and, in severe cases, acute pulmonary edema and life-threatening arrhythmias. Six patients (21%) were asymptomatic, mainly implanted for SSS, with TTS diagnosed on the basis of ECG, echocardiography, and biomarkers. The absence of symptoms at onset did not always predict a benign course; indeed, Wakatsuki et al.^4^ described the case of a female patient implanted for SSS with an initially asymptomatic apical ballooning, who later developed giant negative T-waves and a prolonged corrected QT (QTc) interval leading to polymorphic ventricular tachycardia.

Full recovery of cardiac function was reported in most cases (92%), with varying recovery times (average, 7 weeks); the mortality rate was not negligible (3.6%) and resulted from lethal ventricular arrhythmias or cardiogenic shock. Absent or incomplete recovery of LV function was observed in 8% of patients and could have been the result of either follow-up performed too early or an additional potential harmful effect of RV pacing, as suggested by the fact that the majority of asymptomatic patients had been implanted for SSS (where ventricular pacing is usually avoided), but, unfortunately, the percentage of ventricular pacing was not reported.^2^

Some patients experienced a transient elevation in the RV pacing threshold (especially with bipolar pacing), often associated with the prolongation of QTc interval, probably caused by acute edema and inflammation involving the RV apex, thus increasing local myocardial impedance. The electrical parameters returned to normal values during recovery, along with the recovery of LV function.^5,6^

In a retrospective analysis of a high-volume implantation center, Niewinski et al.^7^ identified nine cases of TTS following pacemaker implantation, with a prevalence of 0.54% on 1655 devices implanted between 2013–2017. In this study, female sex was not predictive of TTS, while cognitive decline and frailty syndrome were independently associated with the risk of TTS occurrence.

We performed an additional literature search and found 13 more case reports that were not included in the systematic review by Strangio et al.,^2^ probably because they did not fulfill the criteria for inclusion. These reports described 13 patients with TTS following pacemaker implantation for AV block (38% men); the correlation between implantation and TTS was less clear because there were other confounding factors, such as the use of isoproterenol before the procedure, concomitant lead extraction, and complications occurring during or shortly after the procedure (ie, pneumothorax, lead dislodgment). Interestingly, in one of these case reports, Scuotto et al.^8^ described a case of TTS occurring after LBB pacing, which is a much more physiological pacing modality compared to RV apical/septal pacing; the patient was a 93-year-old man who developed TTS and cardiogenic shock immediately after an uncomplicated procedure of extraction of an old malfunctioning RV apical lead and concomitant reimplantation of a new lead in the LBB.

Our patient presented with mildly symptomatic complete AV block with narrow QRS and normal LV contractility at baseline; he had several traditional cardiovascular risk factors (hypertension, dyslipidemia, prediabetes, obesity) but did not appear preoperatively to be at high risk for TTS occurrence (due to male sex, no frailty, no cognitive decline, and no history of psychiatric or neurological disorders except for past mild depressive symptoms). The pacemaker implantation was uncomplicated, but it was long and very difficult; moreover, the patient complained of significant pain resulting in physical and emotional stress. Once acute clinical deterioration occurred the next day, the probability of diagnosis was 54.4% as assessed by the InterTAK Diagnostic Score.^1^ Coronary angiography findings were normal, and cardiac perforation and other possible complications were excluded; hence, TTS was considered the most likely diagnosis. It is routine in our center to perform pacemaker and defibrillator implantations under local anesthesia (usually lidocaine 2%) and mild sedation (usually midazolam), so patients are awake during the procedures. In the last 10 years, we have performed about 3000 implants without this kind of complication, even in the case of more stressful and painful procedures. Moreover, acute cardiac deterioration did not occur immediately but rather 24 h after the procedure with 100% paced rhythm.

As it was unclear how long the patient was in complete AV block, it is possible that, as a compensatory response, the peripheral vessels were constricted, and, with the restoration of AV conduction, flash pulmonary edema occurred. However, BP during acute deterioration was not so much greater than that at baseline (160/95 vs. 145/85 mmHg), while apical ballooning and such a marked increase in troponin strongly suggested TTS.

Regarding electrocardiographic aspects, the American College of Cardiology/American Heart Association ST-segment elevation myocardial infarction guidelines offer no formal recommendations for diagnosing acute myocardial infarction (AMI) in RV paced patients, while the European Society of Cardiology guidelines suggest using the Sgarbossa or Smith criteria; the most specific Sgarbossa/Smith criterion is an ST-segment elevation of >5 mm discordant with the QRS complex.^9,10^ In our patient, the first postprocedural ECG **(Figure 2)** showed a 4-mm discordant ST-segment elevation in precordial leads that did not fulfill the criteria for AMI diagnosis.^9,10^ ECG recordings during acute symptoms of TTS showed significant discordant ST-segment elevation from V2–V5 (8 mm in V2 and 6 mm in V3) that fulfilled the Sgarbossa and Smith criteria **(Figure 3)**. Moreover, widespread attenuation of QRS amplitude was evident, especially in lead V6: these changes fulfilled the new Barcelona criteria of an ST deviation of ≥1 mm discordant with QRS in any lead with a maximum R or S voltage of ≤6 mm. The Barcelona criteria were developed for patients with LBB block but have been successfully used in cases of AMI with an RV paced rhythm.^7^

Many questions regarding TTS after pacemaker implantation still remain open, including whether there are predicting factors to risk-stratify patients, but only a multicenter prospective study could provide answers to this and other issues.^2,7,8^ Based on currently available knowledge, despite a benign clinical course in most cases, efforts should be focused on an early diagnosis, also considering a significant percentage of initially asymptomatic cases. An ECG combined with a simple fast echocardiogram before discharge could identify TTS signs, especially in patients deemed at a higher risk of this rare but fearsome complication.

## Conclusions

TTS can be a complication of pacemaker implantation, with some peculiarities compared to the classical form; for example, there is a greater percentage of male and asymptomatic patients. Though its real prevalence is not known, literature data show a case rate of approximately 0.5%, which could be underestimated. Some predictive factors have been identified (frailty and cognitive impairment), but it is still unclear whether peculiar pathophysiologic mechanisms exist and whether the acute initiation of RV pacing is a possible trigger. Several questions remain unanswered—for example, is there a role for active surveillance prior to discharge, even in asymptomatic patients, to minimize the risk of missing this infrequent, yet serious complication? More solid clinical evidence, ideally gathered from prospective studies with long-term follow-up, is needed.

## References

[1] Ghadri JR, Wittstein IS, Prasad A (2018). International expert consensus document on takotsubo syndrome (part I): clinical characteristics, diagnostic criteria, and pathophysiology. Eur Heart J.

[2] Strangio A, Leo I, Sabatino J (2022). Takotsubo syndrome after pacemaker implantation: a systematic review. Rev Cardiovasc Med.

[3] Kurisu S, Inoue I, Kawagoe T (2006). Persistent left ventricular dysfunction in takotsubo cardiomyopathy after pacemaker implantation. Circ J.

[4] Wakatsuki D, Asano T, Mase H, Kurata M, Suzuki H (2020). A case of takotsubo cardiomyopathy developing ventricular fibrillation after a pacemaker implantation. J Cardiol Cases.

[5] Dashwood A, Rahman A, Marashi HA, Jennings C, Raniga M, Dhillon P (2016). Pacemaker-induced takotsubo cardiomyopathy. HeartRhythm Case Rep.

[6] Wei ZH, Dai Q, Wu H, Song J, Wang L, Xu B (2018). Takotsubo cardiomyopathy after pacemaker implantation. J Geriatr Cardiol.

[7] Niewinski P, Walczak T, Królicki T (2020). Frailty and cognitive impairment are predictive of takotsubo syndrome following pacemaker implantation. Pacing Clin Electrophysiol.

[8] Scuotto F, Albertini CMM, Lemos SGD, Staico R, Assad RS, Cirenza C (2021). Takotsubo cardiomyopathy after left bundle branch pacing: a case report. HeartRhythm Case Rep.

[9] Ramanathan RR, Rangaswamy VV, Nanda Kumar T (2023). A case of acute myocardial infarction in paced rhythm. Utility of the Barcelona algorithm. J Electrocardiol.

[10] Dodd KW, Zvosec DL, Hart MA (2021). Electrocardiographic diagnosis of acute coronary occlusion myocardial infarction in ventricular paced rhythm using the modified Sgarbossa criteria. Ann Emerg Med.

